# *N*-(2-Chloro­phen­yl)-4-methyl­benzamide

**DOI:** 10.1107/S1600536811038840

**Published:** 2011-09-30

**Authors:** Vinola Z. Rodrigues, Peter Herich, B. Thimme Gowda, Jozef Kožíšek

**Affiliations:** a Department of Chemistry, Mangalore University, Mangalagangotri 574 199, Mangalore, India; b Institute of Physical Chemistry and Chemical Physics, Slovak University of Technology, Radlinského 9, SK-812 37 Bratislava, Slovak Republic

## Abstract

The asymmetric unit of the title compound, C_14_H_12_ClNO, contains two independent mol­ecules in which the dihedral angles between the two aromatic rings are 51.76 (6) and 51.48 (7)°. The crystal structure is stabilized by inter­molecular N—H⋯O hydrogen bonds, which link the mol­ecules into chains running along the *c* axis.

## Related literature

For preparation of the title compound, see: Gowda *et al.* (2003 ▶). For our studies on the effects of substituents on the structures and other aspects of *N*-(ar­yl)-amides, see: Arjunan *et al.* (2004 ▶); Bowes *et al.* (2003 ▶); Gowda *et al.* (2001 ▶); Rodrigues *et al.* (2011 ▶); Saeed *et al.* (2010 ▶) on *N*-(ar­yl)-methane­sulfonamides, see: Gowda *et al.* (2007 ▶) and on *N*-(ar­yl)-aryl­sulfonamides, see: Gowda *et al.* (2005 ▶). 
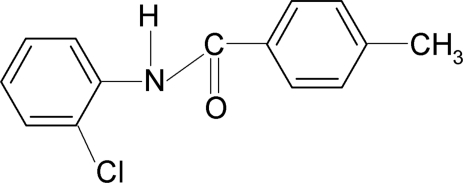


## Experimental

### 

#### Crystal data

C_14_H_12_ClNO
*M* *_r_* = 245.70
Monoclinic, 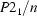

*a* = 9.6940 (5) Å
*b* = 27.4495 (9) Å
*c* = 9.9025 (4) Å
β = 106.730 (5)°
*V* = 2523.48 (19) Å^3^
*Z* = 8
Mo *K*α radiation
μ = 0.29 mm^−1^
*T* = 293 K
0.97 × 0.13 × 0.10 mm

#### Data collection

Oxford Diffraction Xcalibur Ruby Gemini diffractometer
Absorption correction: analytical [*CrysAlis RED* (Oxford Diffraction, 2009 ▶) based on expressions derived by Clark & Reid (1995 ▶)] *T*_min_ = 0.957, *T*_max_ = 0.972
47577 measured reflections
7045 independent reflections
2850 reflections with *I* > 2σ(*I*)
*R*_int_ = 0.046

#### Refinement

*R*[*F* ^2^ > 2σ(*F* ^2^)] = 0.043
*wR*(*F* ^2^) = 0.126
*S* = 0.85
7045 reflections
307 parameters
H-atom parameters constrained
Δρ_max_ = 0.24 e Å^−3^
Δρ_min_ = −0.30 e Å^−3^

### 

Data collection: *CrysAlis CCD* (Oxford Diffraction, 2009 ▶); cell refinement: *CrysAlis CCD*; data reduction: *CrysAlis RED* (Oxford Diffraction, 2009 ▶); program(s) used to solve structure: *SHELXS97* (Sheldrick, 2008 ▶); program(s) used to refine structure: *SHELXL97* (Sheldrick, 2008 ▶); molecular graphics: *DIAMOND* (Brandenburg, 2002 ▶); software used to prepare material for publication: *enCIFer* (Allen *et al.*, 2004 ▶).

## Supplementary Material

Crystal structure: contains datablock(s) I, global. DOI: 10.1107/S1600536811038840/bt5650sup1.cif

Structure factors: contains datablock(s) I. DOI: 10.1107/S1600536811038840/bt5650Isup2.hkl

Supplementary material file. DOI: 10.1107/S1600536811038840/bt5650Isup3.cml

Additional supplementary materials:  crystallographic information; 3D view; checkCIF report

## Figures and Tables

**Table 1:** Hydrogen-bond geometry (Å, °)

*D*—H⋯*A*	*D*—H	H⋯*A*	*D*⋯*A*	*D*—H⋯*A*
N15—H15*A*⋯O34	0.86	2.02	2.8408 (16)	159
N32—H32*A*⋯O17^i^	0.86	2.01	2.8455 (16)	165

Symmetry code: (i) 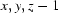.

**Fig. 1.**
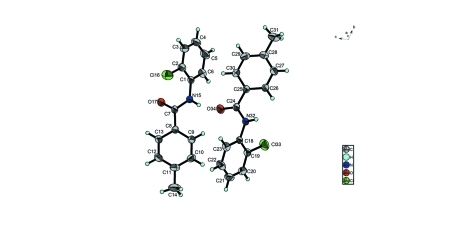
Molecular structure of the title compound showing the atom labelling scheme. Displacement ellipsoids are drawn at the 50% probability level. H atoms are represented as small spheres of arbitrary radii.

**Fig. 2.**
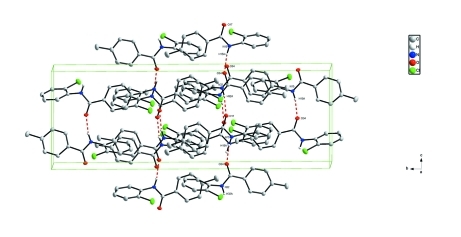
Part of the crystal structure of the title compound. Molecular chains are generated by N—H···O hydrogen bonds which are shown by dashed lines. H atoms not involved in intermolecular bonding have been omitted.

## supplementary crystallographic information

### Comment

The amide and sulfonamide moieties are the constituents of many biologically significant compounds. As part of our work on the substituent effects on the structures and other aspects of *N*-(aryl)-amides (Arjunan *et al.*, 2004; Bowes *et al.*, 2003; Gowda *et al.*, 2001; Saeed *et al.*, 2010; Rodrigues *et al.*, 2011), *N*-(aryl)-methanesulfonamides (Gowda *et al.*, 2007) and *N*-(aryl)-arylsulfonamides (Gowda *et al.*, 2005), in the present work, the crystal structure of *N*-(2-Chlorophenyl)-4-methylbenzamide (I) has been determined (Fig.1). The asymmetric unit of (I) contains two independent molecules. In the crystal, the *ortho*-Cl substituent in the anilino ring is positioned *syn* to the N–H bond in one of the molecules and *anti* in the other molecule. Further, the N—H and C=O bonds in the C—NH—C(O)—C segment are *anti* to each other in both the molecules, similar to that observed in *N*-(2-methylphenyl)- 4-methylbenzamide (II)(Rodrigues *et al.*, 2011).

The packing of molecules linked by N—H···O hydrogen bonds into infinite chains is shown in Fig. 2.

### Experimental

The title compound was prepared according to the method described by Gowda *et al.* (2003). The purity of the compound was checked by determining its melting point. It was characterized by recording its infrared and NMR spectra. cuboid-like colourless single crystals of the title compound were obtained by slow evaporation from an ethanol solution of the compound (0.5 g in about 30 ml of ethanol) at room temperature.

### Refinement

All H atoms were visible in difference maps and then treated as riding atoms with C–H distances of 0.93Å (C-aromatic), 0.96Å (*C*-methyl) and N—H = 0.86 Å. The *U*_iso_(H) values were set at 1.2 *U*_eq_(C-aromatic, N) and 1.5 *U*_eq_(C-methyl).

### Crystal data

**Table:**

C_14_H_12_ClNO	*F*(000) = 1024
*M**_r_* = 245.70	*D*_x_ = 1.293 Mg m^−^^3^
Monoclinic, *P*2_1_/*n*	Mo *K*α radiation, λ = 0.71073 Å
Hall symbol: -P 2yn	Cell parameters from 13361 reflections
*a* = 9.6940 (5) Å	θ = 3.6–29.4°
*b* = 27.4495 (9) Å	µ = 0.29 mm^−^^1^
*c* = 9.9025 (4) Å	*T* = 293 K
β = 106.730 (5)°	Cuboid, colorless
*V* = 2523.48 (19) Å^3^	0.97 × 0.13 × 0.10 mm
*Z* = 8	

### Data collection

**Table:**

Oxford Diffraction Xcalibur Ruby Gemini diffractometer	7045 independent reflections
Radiation source: fine-focus sealed tube	2850 reflections with *I* > 2σ(*I*)
graphite	*R*_int_ = 0.046
Detector resolution: 10.4340 pixels mm^-1^	θ_max_ = 29.4°, θ_min_ = 3.6°
ω scans	*h* = −13→13
Absorption correction: analytical [*CrysAlis RED* (Oxford Diffraction, 2009) based on expressions derived by Clark & Reid (1995)]	*k* = −37→37
*T*_min_ = 0.957, *T*_max_ = 0.972	*l* = −13→12
47577 measured reflections	

### Refinement

**Table:**

Refinement on *F*^2^	Primary atom site location: structure-invariant direct methods
Least-squares matrix: full	Secondary atom site location: difference Fourier map
*R*[*F*^2^ > 2σ(*F*^2^)] = 0.043	Hydrogen site location: inferred from neighbouring sites
*wR*(*F*^2^) = 0.126	H-atom parameters constrained
*S* = 0.85	*w* = 1/[σ^2^(*F*_o_^2^) + (0.0704*P*)^2^] where *P* = (*F*_o_^2^ + 2*F*_c_^2^)/3
7045 reflections	(Δ/σ)_max_ < 0.001
307 parameters	Δρ_max_ = 0.24 e Å^−^^3^
0 restraints	Δρ_min_ = −0.30 e Å^−^^3^

### Special details

**Table:**

Geometry. All e.s.d.'s (except the e.s.d. in the dihedral angle between two l.s. planes) are estimated using the full covariance matrix. The cell e.s.d.'s are taken into account individually in the estimation of e.s.d.'s in distances, angles and torsion angles; correlations between e.s.d.'s in cell parameters are only used when they are defined by crystal symmetry. An approximate (isotropic) treatment of cell e.s.d.'s is used for estimating e.s.d.'s involving l.s. planes.
Refinement. Refinement of *F*^2^ against ALL reflections. The weighted *R*-factor *wR* and goodness of fit *S* are based on *F*^2^, conventional *R*-factors *R* are based on *F*, with *F* set to zero for negative *F*^2^. The threshold expression of *F*^2^ > σ(*F*^2^) is used only for calculating *R*-factors(gt) *etc*. and is not relevant to the choice of reflections for refinement. *R*-factors based on *F*^2^ are statistically about twice as large as those based on *F*, and *R*- factors based on ALL data will be even larger.

### Fractional atomic coordinates and isotropic or equivalent isotropic displacement parameters (Å^2^)

**Table:**

	*x*	*y*	*z*	*U*_iso_*/*U*_eq_	
C1	0.4465 (2)	0.18661 (6)	0.78372 (15)	0.0492 (4)	
C2	0.5890 (2)	0.19536 (7)	0.85616 (18)	0.0567 (5)	
C3	0.6412 (3)	0.24255 (8)	0.8811 (2)	0.0712 (6)	
H3A	0.7374	0.2481	0.9296	0.085*	
C4	0.5494 (3)	0.28101 (8)	0.8336 (2)	0.0794 (7)	
H4A	0.5839	0.3128	0.8495	0.095*	
C5	0.4082 (3)	0.27296 (8)	0.7632 (2)	0.0827 (7)	
H5A	0.3463	0.2991	0.7320	0.099*	
C6	0.3572 (2)	0.22594 (7)	0.7386 (2)	0.0676 (5)	
H6A	0.2608	0.2207	0.6906	0.081*	
C7	0.3647 (2)	0.10713 (6)	0.83990 (16)	0.0497 (4)	
C8	0.30994 (18)	0.05833 (6)	0.78704 (16)	0.0466 (4)	
C9	0.2405 (2)	0.04866 (7)	0.64638 (17)	0.0555 (5)	
H9A	0.2249	0.0738	0.5808	0.067*	
C10	0.1947 (2)	0.00242 (7)	0.6028 (2)	0.0653 (5)	
H10A	0.1467	−0.0031	0.5083	0.078*	
C11	0.2182 (2)	−0.03609 (7)	0.6964 (2)	0.0677 (5)	
C12	0.2852 (2)	−0.02635 (7)	0.8368 (2)	0.0693 (6)	
H12A	0.3009	−0.0516	0.9021	0.083*	
C13	0.3290 (2)	0.02018 (7)	0.88200 (18)	0.0583 (5)	
H13A	0.3719	0.0260	0.9774	0.070*	
C14	0.1746 (4)	−0.08743 (8)	0.6468 (3)	0.1105 (10)	
H14C	0.1994	−0.1092	0.7260	0.133*	
H14B	0.2242	−0.0971	0.5800	0.133*	
H14A	0.0725	−0.0886	0.6028	0.133*	
C18	0.3176 (2)	0.05619 (6)	0.25095 (15)	0.0472 (4)	
C19	0.1723 (2)	0.04811 (6)	0.18847 (17)	0.0556 (5)	
C20	0.1129 (2)	0.00237 (8)	0.1859 (2)	0.0703 (6)	
H20A	0.0150	−0.0025	0.1435	0.084*	
C21	0.1995 (3)	−0.03562 (8)	0.2465 (2)	0.0755 (6)	
H21A	0.1605	−0.0667	0.2437	0.091*	
C22	0.3426 (3)	−0.02856 (7)	0.3110 (2)	0.0755 (6)	
H22A	0.4003	−0.0546	0.3535	0.091*	
C23	0.4020 (2)	0.01741 (7)	0.31306 (19)	0.0624 (5)	
H23A	0.4997	0.0221	0.3568	0.075*	
C24	0.41322 (19)	0.13420 (6)	0.35262 (16)	0.0511 (4)	
C25	0.44828 (19)	0.18501 (6)	0.32278 (15)	0.0476 (4)	
C26	0.4024 (2)	0.20526 (6)	0.18911 (17)	0.0621 (5)	
H26A	0.3556	0.1858	0.1129	0.074*	
C27	0.4250 (2)	0.25370 (7)	0.1674 (2)	0.0683 (6)	
H27A	0.3930	0.2663	0.0765	0.082*	
C28	0.4937 (2)	0.28404 (7)	0.2766 (2)	0.0618 (5)	
C29	0.5419 (2)	0.26368 (7)	0.4093 (2)	0.0707 (6)	
H29A	0.5907	0.2832	0.4848	0.085*	
C30	0.5195 (2)	0.21513 (7)	0.43291 (18)	0.0606 (5)	
H30A	0.5526	0.2025	0.5237	0.073*	
C31	0.5128 (3)	0.33727 (7)	0.2505 (3)	0.0915 (7)	
H31C	0.5625	0.3529	0.3377	0.110*	
H31B	0.4202	0.3522	0.2124	0.110*	
H31A	0.5681	0.3407	0.1847	0.110*	
N15	0.39382 (16)	0.13853 (5)	0.74748 (13)	0.0507 (4)	
H15A	0.3799	0.1290	0.6618	0.061*	
N32	0.37672 (17)	0.10304 (5)	0.24535 (13)	0.0534 (4)	
H32A	0.3900	0.1120	0.1668	0.064*	
O17	0.38313 (17)	0.11887 (5)	0.96324 (11)	0.0771 (5)	
O34	0.4147 (2)	0.12111 (5)	0.47127 (12)	0.0924 (5)	
Cl16	0.70621 (7)	0.14715 (2)	0.91490 (7)	0.0936 (2)	
Cl33	0.06234 (7)	0.09610 (2)	0.10827 (7)	0.0956 (2)	

### Atomic displacement parameters (Å^2^)

**Table:**

	*U*^11^	*U*^22^	*U*^33^	*U*^12^	*U*^13^	*U*^23^
C1	0.0709 (13)	0.0453 (10)	0.0353 (8)	−0.0042 (9)	0.0213 (8)	0.0003 (7)
C2	0.0676 (14)	0.0507 (11)	0.0551 (10)	−0.0015 (10)	0.0228 (9)	−0.0006 (8)
C3	0.0802 (15)	0.0639 (14)	0.0698 (13)	−0.0166 (12)	0.0220 (11)	−0.0058 (10)
C4	0.114 (2)	0.0492 (13)	0.0813 (14)	−0.0168 (14)	0.0383 (15)	−0.0030 (11)
C5	0.105 (2)	0.0487 (13)	0.0948 (16)	0.0094 (13)	0.0286 (15)	0.0157 (11)
C6	0.0756 (14)	0.0593 (13)	0.0651 (12)	0.0025 (11)	0.0155 (10)	0.0129 (10)
C7	0.0668 (12)	0.0505 (10)	0.0354 (9)	−0.0018 (9)	0.0203 (8)	−0.0013 (7)
C8	0.0533 (11)	0.0490 (10)	0.0408 (9)	−0.0024 (8)	0.0187 (7)	−0.0022 (8)
C9	0.0668 (12)	0.0555 (11)	0.0452 (9)	−0.0073 (9)	0.0175 (9)	−0.0013 (8)
C10	0.0708 (14)	0.0716 (14)	0.0548 (11)	−0.0152 (11)	0.0202 (9)	−0.0157 (10)
C11	0.0751 (14)	0.0558 (12)	0.0801 (14)	−0.0189 (10)	0.0351 (11)	−0.0147 (11)
C12	0.0872 (16)	0.0539 (12)	0.0719 (13)	−0.0079 (11)	0.0309 (11)	0.0086 (10)
C13	0.0735 (13)	0.0547 (12)	0.0478 (9)	−0.0055 (10)	0.0192 (9)	0.0040 (9)
C14	0.145 (3)	0.0753 (16)	0.122 (2)	−0.0446 (17)	0.0568 (19)	−0.0319 (15)
C18	0.0676 (13)	0.0425 (10)	0.0359 (8)	−0.0033 (9)	0.0218 (8)	−0.0009 (7)
C19	0.0702 (14)	0.0515 (11)	0.0455 (9)	−0.0003 (10)	0.0172 (9)	0.0017 (8)
C20	0.0780 (15)	0.0693 (14)	0.0619 (12)	−0.0175 (12)	0.0171 (10)	−0.0064 (10)
C21	0.106 (2)	0.0494 (12)	0.0799 (14)	−0.0162 (13)	0.0409 (14)	−0.0102 (11)
C22	0.103 (2)	0.0470 (13)	0.0871 (14)	0.0161 (12)	0.0443 (14)	0.0128 (10)
C23	0.0660 (13)	0.0630 (13)	0.0610 (11)	0.0055 (11)	0.0228 (9)	0.0065 (9)
C24	0.0676 (12)	0.0538 (11)	0.0357 (9)	−0.0019 (9)	0.0210 (8)	−0.0024 (8)
C25	0.0581 (11)	0.0484 (10)	0.0398 (9)	−0.0006 (8)	0.0194 (8)	−0.0032 (8)
C26	0.0951 (16)	0.0470 (11)	0.0432 (10)	−0.0061 (10)	0.0185 (9)	−0.0058 (8)
C27	0.1027 (17)	0.0506 (12)	0.0544 (11)	−0.0028 (11)	0.0273 (11)	0.0041 (9)
C28	0.0671 (13)	0.0489 (11)	0.0769 (13)	−0.0062 (10)	0.0326 (10)	−0.0050 (10)
C29	0.0736 (15)	0.0660 (13)	0.0696 (13)	−0.0172 (11)	0.0159 (11)	−0.0206 (11)
C30	0.0692 (13)	0.0615 (12)	0.0469 (10)	−0.0078 (10)	0.0100 (9)	−0.0059 (9)
C31	0.109 (2)	0.0522 (13)	0.1262 (19)	−0.0105 (13)	0.0550 (16)	−0.0083 (13)
N15	0.0737 (10)	0.0472 (8)	0.0339 (7)	−0.0094 (7)	0.0200 (7)	−0.0038 (6)
N32	0.0805 (11)	0.0490 (9)	0.0340 (7)	−0.0119 (7)	0.0215 (7)	−0.0006 (6)
O17	0.1376 (14)	0.0618 (8)	0.0409 (7)	−0.0250 (8)	0.0399 (7)	−0.0084 (6)
O34	0.1802 (17)	0.0653 (9)	0.0414 (7)	−0.0260 (10)	0.0475 (9)	−0.0063 (6)
Cl16	0.0746 (4)	0.0705 (4)	0.1285 (5)	0.0101 (3)	0.0178 (3)	0.0039 (3)
Cl33	0.0833 (4)	0.0885 (4)	0.1050 (5)	0.0137 (3)	0.0112 (3)	0.0323 (3)

### Geometric parameters (Å, °)

**Table:**

C1—C6	1.375 (3)	C18—C19	1.384 (3)
C1—C2	1.382 (3)	C18—N32	1.415 (2)
C1—N15	1.423 (2)	C19—C20	1.379 (3)
C2—C3	1.387 (3)	C19—Cl33	1.7365 (19)
C2—Cl16	1.732 (2)	C20—C21	1.365 (3)
C3—C4	1.374 (3)	C20—H20A	0.9300
C3—H3A	0.9300	C21—C22	1.364 (3)
C4—C5	1.364 (3)	C21—H21A	0.9300
C4—H4A	0.9300	C22—C23	1.385 (3)
C5—C6	1.378 (3)	C22—H22A	0.9300
C5—H5A	0.9300	C23—H23A	0.9300
C6—H6A	0.9300	C24—O34	1.2248 (17)
C7—O17	1.2255 (17)	C24—N32	1.330 (2)
C7—N15	1.345 (2)	C24—C25	1.485 (2)
C7—C8	1.480 (2)	C25—C30	1.384 (2)
C8—C13	1.384 (2)	C25—C26	1.386 (2)
C8—C9	1.387 (2)	C26—C27	1.374 (3)
C9—C10	1.373 (3)	C26—H26A	0.9300
C9—H9A	0.9300	C27—C28	1.375 (3)
C10—C11	1.381 (3)	C27—H27A	0.9300
C10—H10A	0.9300	C28—C29	1.380 (3)
C11—C12	1.380 (3)	C28—C31	1.505 (3)
C11—C14	1.512 (3)	C29—C30	1.381 (3)
C12—C13	1.379 (3)	C29—H29A	0.9300
C12—H12A	0.9300	C30—H30A	0.9300
C13—H13A	0.9300	C31—H31C	0.9600
C14—H14C	0.9600	C31—H31B	0.9600
C14—H14B	0.9600	C31—H31A	0.9600
C14—H14A	0.9600	N15—H15A	0.8600
C18—C23	1.375 (2)	N32—H32A	0.8600
			
C6—C1—C2	118.26 (17)	C20—C19—C18	121.37 (18)
C6—C1—N15	119.97 (17)	C20—C19—Cl33	118.95 (17)
C2—C1—N15	121.63 (16)	C18—C19—Cl33	119.67 (14)
C1—C2—C3	120.95 (18)	C21—C20—C19	119.1 (2)
C1—C2—Cl16	120.17 (14)	C21—C20—H20A	120.4
C3—C2—Cl16	118.88 (17)	C19—C20—H20A	120.4
C4—C3—C2	119.3 (2)	C22—C21—C20	120.8 (2)
C4—C3—H3A	120.3	C22—C21—H21A	119.6
C2—C3—H3A	120.3	C20—C21—H21A	119.6
C5—C4—C3	120.4 (2)	C21—C22—C23	119.9 (2)
C5—C4—H4A	119.8	C21—C22—H22A	120.0
C3—C4—H4A	119.8	C23—C22—H22A	120.0
C4—C5—C6	119.9 (2)	C18—C23—C22	120.5 (2)
C4—C5—H5A	120.1	C18—C23—H23A	119.8
C6—C5—H5A	120.1	C22—C23—H23A	119.8
C1—C6—C5	121.2 (2)	O34—C24—N32	120.29 (15)
C1—C6—H6A	119.4	O34—C24—C25	121.75 (14)
C5—C6—H6A	119.4	N32—C24—C25	117.94 (13)
O17—C7—N15	120.71 (15)	C30—C25—C26	117.58 (16)
O17—C7—C8	121.67 (14)	C30—C25—C24	119.67 (14)
N15—C7—C8	117.62 (13)	C26—C25—C24	122.55 (15)
C13—C8—C9	117.97 (16)	C27—C26—C25	121.04 (16)
C13—C8—C7	118.71 (14)	C27—C26—H26A	119.5
C9—C8—C7	123.32 (15)	C25—C26—H26A	119.5
C10—C9—C8	120.80 (17)	C26—C27—C28	121.73 (17)
C10—C9—H9A	119.6	C26—C27—H27A	119.1
C8—C9—H9A	119.6	C28—C27—H27A	119.1
C9—C10—C11	121.40 (17)	C27—C28—C29	117.26 (17)
C9—C10—H10A	119.3	C27—C28—C31	120.59 (19)
C11—C10—H10A	119.3	C29—C28—C31	122.15 (19)
C12—C11—C10	117.82 (17)	C28—C29—C30	121.68 (17)
C12—C11—C14	121.0 (2)	C28—C29—H29A	119.2
C10—C11—C14	121.2 (2)	C30—C29—H29A	119.2
C13—C12—C11	121.17 (17)	C29—C30—C25	120.69 (17)
C13—C12—H12A	119.4	C29—C30—H30A	119.7
C11—C12—H12A	119.4	C25—C30—H30A	119.7
C12—C13—C8	120.78 (17)	C28—C31—H31C	109.5
C12—C13—H13A	119.6	C28—C31—H31B	109.5
C8—C13—H13A	119.6	H31C—C31—H31B	109.5
C11—C14—H14C	109.5	C28—C31—H31A	109.5
C11—C14—H14B	109.5	H31C—C31—H31A	109.5
H14C—C14—H14B	109.5	H31B—C31—H31A	109.5
C11—C14—H14A	109.5	C7—N15—C1	123.57 (12)
H14C—C14—H14A	109.5	C7—N15—H15A	118.2
H14B—C14—H14A	109.5	C1—N15—H15A	118.2
C23—C18—C19	118.30 (16)	C24—N32—C18	124.80 (13)
C23—C18—N32	121.70 (17)	C24—N32—H32A	117.6
C19—C18—N32	119.96 (16)	C18—N32—H32A	117.6
			
C6—C1—C2—C3	−0.8 (2)	C18—C19—C20—C21	0.0 (3)
N15—C1—C2—C3	174.95 (14)	Cl33—C19—C20—C21	178.76 (15)
C6—C1—C2—Cl16	−179.78 (13)	C19—C20—C21—C22	1.1 (3)
N15—C1—C2—Cl16	−4.0 (2)	C20—C21—C22—C23	−1.3 (3)
C1—C2—C3—C4	0.3 (3)	C19—C18—C23—C22	0.9 (2)
Cl16—C2—C3—C4	179.27 (15)	N32—C18—C23—C22	−176.82 (15)
C2—C3—C4—C5	0.4 (3)	C21—C22—C23—C18	0.3 (3)
C3—C4—C5—C6	−0.5 (3)	O34—C24—C25—C30	17.0 (3)
C2—C1—C6—C5	0.7 (3)	N32—C24—C25—C30	−164.36 (17)
N15—C1—C6—C5	−175.18 (16)	O34—C24—C25—C26	−157.66 (19)
C4—C5—C6—C1	0.0 (3)	N32—C24—C25—C26	21.0 (3)
O17—C7—C8—C13	−23.7 (3)	C30—C25—C26—C27	−1.0 (3)
N15—C7—C8—C13	156.29 (17)	C24—C25—C26—C27	173.79 (19)
O17—C7—C8—C9	156.49 (18)	C25—C26—C27—C28	0.0 (3)
N15—C7—C8—C9	−23.5 (3)	C26—C27—C28—C29	1.2 (3)
C13—C8—C9—C10	−1.2 (3)	C26—C27—C28—C31	−178.1 (2)
C7—C8—C9—C10	178.60 (17)	C27—C28—C29—C30	−1.5 (3)
C8—C9—C10—C11	−1.2 (3)	C31—C28—C29—C30	177.8 (2)
C9—C10—C11—C12	2.3 (3)	C28—C29—C30—C25	0.5 (3)
C9—C10—C11—C14	−176.7 (2)	C26—C25—C30—C29	0.7 (3)
C10—C11—C12—C13	−0.9 (3)	C24—C25—C30—C29	−174.21 (18)
C14—C11—C12—C13	178.0 (2)	O17—C7—N15—C1	−0.2 (3)
C11—C12—C13—C8	−1.4 (3)	C8—C7—N15—C1	179.78 (16)
C9—C8—C13—C12	2.5 (3)	C6—C1—N15—C7	−107.11 (19)
C7—C8—C13—C12	−177.30 (18)	C2—C1—N15—C7	77.2 (2)
C23—C18—C19—C20	−1.0 (2)	O34—C24—N32—C18	9.7 (3)
N32—C18—C19—C20	176.71 (15)	C25—C24—N32—C18	−168.96 (16)
C23—C18—C19—Cl33	−179.72 (12)	C23—C18—N32—C24	−77.9 (2)
N32—C18—C19—Cl33	−2.0 (2)	C19—C18—N32—C24	104.46 (19)

### Hydrogen-bond geometry (Å, °)

**Table:**

*D*—H···*A*	*D*—H	H···*A*	*D*···*A*	*D*—H···*A*
N15—H15A···O34	0.86	2.02	2.8408 (16)	159.
N32—H32A···O17^i^	0.86	2.01	2.8455 (16)	165.

Symmetry codes: (i) *x*, *y*, *z*−1.
